# IRF8 deficiency-induced myeloid-derived suppressor cell promote immune evasion in lung adenocarcinoma

**DOI:** 10.1186/s12967-024-05519-7

**Published:** 2024-07-24

**Authors:** Zhen Gao, Shang Liu, Han Xiao, Meng Li, Wan-gang Ren, Lin Xu, Zhong-min Peng

**Affiliations:** Department of Thoracic Surgery, Provincial Hospital Affiliated to Shandong First Medical University, Shandong First Medical University, Jinan, 250021 Shandong People’s Republic of China

**Keywords:** Lung adenocarcinoma, MDSCs, IRF8, Tumor microenvironment, Immune escape

## Abstract

**Background:**

Patients with lung adenocarcinoma (LUAD) have a low response rate to immune checkpoint blockade. It is highly important to explore the tumor immune escape mechanism of LUAD patients and expand the population of patients who may benefit from immunotherapy.

**Methods:**

Based on 954 bulk RNA-seq data of LUAD patients and 15 single-cell RNA-seq data, the relationships between tumor immune dysfunction and exclusion (TIDE) scores and survival prognosis in each patient were calculated and evaluated, and the immune escape mechanism affecting the independent prognosis of LUAD patients was identified. Functional enrichment analysis explored the antitumour immune response and biological behavior of tumor cells among different LUAD groups. Single-cell annotation and pseudotemporal analysis were used to explore the target molecules and immune escape mechanisms of LUAD.

**Results:**

Myeloid-derived suppressor cells (MDSCs) and IRF8 were identified as risk and protective factors for the independent prognosis of LUAD patients, respectively. In the tumor microenvironment of patients with high infiltration of MDSCs, the antitumor immune response is significantly suppressed, while tumor cell division, proliferation, and distant metastasis are significantly enhanced. Single-cell RNA-seq analysis revealed that IRF8 is an important regulator of MDSC differentiation in LUAD myeloid cells. In addition, IRF8 may regulate the differentiation of MDSCs through the IL6-JAK-STAT3 signalling pathway.

**Conclusions:**

IRF8 deficiency impairs the normal development of LUAD myeloid cells and induces their differentiation into MDSCs, thereby accelerating the immune escape of LUAD cells. IRF8-targeted activation to inhibit the formation of MDSCs may be a new target for immunotherapy in LUAD.

**Supplementary Information:**

The online version contains supplementary material available at 10.1186/s12967-024-05519-7.

## Introduction

Lung cancer has the leading mortality rate and the second leading incidence among all cancers [1], and adenocarcinoma is the most common subtype of lung cancer [2]. In the past decade, significant advances have been made in the treatment of lung adenocarcinoma, which has greatly improved survival rates [3]. In particular, in first-line or second-line treatment strategies, the development of specific antibodies targeting programmed death 1 (PD-1) and programmed death ligand 1 (PD-L1) has resulted in unprecedented prolongation of survival time for some lung adenocarcinoma patients [4]. Although PD-1/PD-L1 blockade has achieved significant effects in clinical practice, the response rate in patients with nonselective non-small cell lung cancer is approximately 20%, and the response rate in patients with lung adenocarcinoma is even lower [5]. Therefore, it is highly important to explore the tumor immune mechanism of lung adenocarcinoma and expand the population of patients who benefit from immunotherapy.

Recent studies have revealed that there are two different immune mechanisms involved in tumor immune escape [6, 7]. Although there is high infiltration of cytotoxic T cells in the tumor microenvironment (TME) of some tumors, these T cells are often in a functionally exhausted state and lose their ability to kill tumors. In other tumors, the presence of immune suppressive cells, such as cancer-associated fibroblasts (CAFs), myeloid-derived suppressor cells (MDSCs), and the M2 subtype of tumor-associated macrophages (M2), restricts T-cell infiltration into the TME. However, PD-1/PD-L1 blockade in the TME with T-cell exhaustion and/or T-cell dysfunction does not provide good tumor immune suppression [8]. Hence, elucidating the main immune escape mechanism of lung adenocarcinoma can greatly reduce the risk of PD-1/PD-L1 blockade, identify new immune targets and broaden the therapeutic scope of immune checkpoint inhibitors. Based on the two main mechanisms of tumor immune evasion, Peng, J. team constructed an algorithm framework for tumor immune dysfunction and exclusion (TIDE) based on the genomic characteristics of T-cell dysfunction and T-cell exclusion (CAFs, MDSCs, and M2) [8]. The TIDE framework can calculate T-cell dysfunction and T-cell exclusion scores as well as CAF, MDSC, and M2 infiltration levels in the TME. This study provides a feasible method and practical basis for exploring the immune escape mechanism of lung adenocarcinoma.

An increasing number of studies have shown that the genomic and transcriptome characteristics of tumors can help researchers explore and predict immune processes and antitumour immune responses in the TME. For example, a higher tumor mutation burden, high PD-L1 expression, and tumor antigen quality are associated with clinical benefits from immunotherapy [9–12]. Ideally, many tumor molecular profiles and patient clinical outcomes can be used to assess TME immune responses and levels of cellular infiltration. For instance, a recent analysis of TCGA and PRECOG data revealed that the level of tumor infiltration of different immune cell types significantly impacts the overall survival rate of patients [13–15]. However, the above analysis can be difficult to perform using laboratory methods. At present, emerging single-cell RNA-seq technology can be used to characterize diseases at the cellular level. From the perspective of the cell atlas, it can identify the specificity of different cell types and differences between cells and explore the cell development trajectory and synergistic molecules. Thus, based on the valuable resources of the public tumor molecular spectrum, we combined single-cell RNA-seq and bulk RNA-seq to explore the main immune escape mechanisms and tumor immune-related molecular profiles of lung adenocarcinoma.

In this study, we integrated and modelled data from 6 human lung adenocarcinomas, comprising a total of 954 transcripts and 15 single-cell transcripts from 79078 cells. Based on the TIDE framework, T-cell dysfunction and T-cell exclusion scores, as well as CAF, MDSC, and M2 infiltration levels, were evaluated in each patient to identify the main immune escape mechanisms affecting the survival and prognosis of patients with lung adenocarcinoma. The synergistic molecules involved in tumor immune escape in lung adenocarcinoma patients were explored and identified through single-cell RNA sequencing. These findings may provide a new valuable idea for the immunotherapy of lung adenocarcinoma. These findings may provide a new valuable idea for the immunotherapy of lung adenocarcinoma.

## Materials and methods

### Patients

**Fig. 1:**
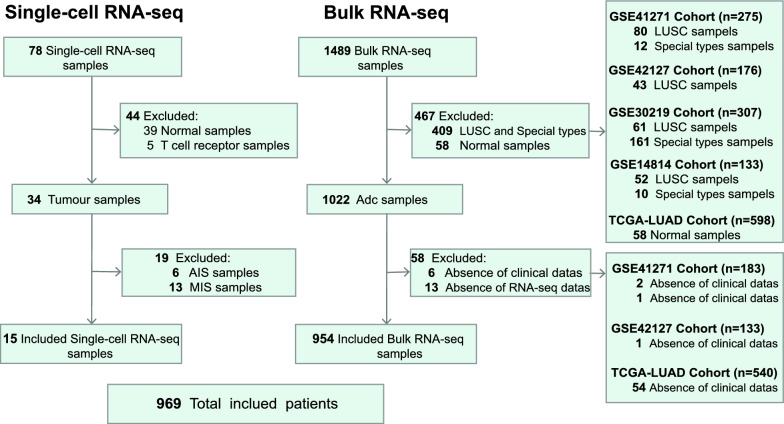
Flow diagram: the patient selection and exclusion criteria

This study obtained transcriptomic sequencing data for lung adenocarcinoma from the GEO (GSE41271, GSE42127, GSE30219, and GSE14814) and TCGA (LUAD) databases. Among them, the GSE41271 and TCGA cohorts were the main study cohorts, and the GSE42127, GSE30219, and GSE14814 cohorts were the validation cohorts. This study obtained lung adenocarcinoma single-cell RNA sequencing data from the GEO (GSE164789) database. Based on the inclusion and exclusion criteria detailed in Fig. 1, 954 patients were included in the study, including lung adenocarcinoma transcriptome samples with complete clinical information from GSE41271 (180 patients), GSE42127 (132 patients), GSE30219 (85 patients), GSE14814 (71 patients), and TCGA-LUAD (486 patients), as well as 15 lung adenocarcinoma single-cell RNA sequencing samples. All transcriptome data were converted into transcripts per million reads (TPM) format, and log2 (TPM + 1) was transformed for subsequent data analysis. The clinical information of the lung adenocarcinoma patients included age, sex, smoking history, pathological stage, overall survival time, and survival status. The GSE30219 and GSE14814 cohorts are early-stage LUAD cohorts (pathological stages: I-II).

### Tumor immune dysfunction and exclusion (TIDE) analysis

There are two different immune escape mechanisms in tumors: on the one hand, some immunosuppressive factors can prevent the infiltration of T cells; on the other hand, some tumors have high cytotoxicity. T cells infiltrate the level, but these T cells are in a functionally inactive state. TIDE (http://tide.dfci.harvard.edu) predicts the immune escape ability of tumors by comprehensively evaluating the activity of these two mechanisms. Based on the TIDE framework, the T-cell exclusion score, MDSC infiltration score, M2 infiltration score, CAF infiltration score, and T-cell dysfunction score were calculated for each lung adenocarcinoma sample.

### Survival analysis

K‒M survival curves were used to show the changes in survival rates among different groups, and log-rank tests were used to assess the significance of differences in survival rates between groups. Univariate Cox regression was used for correlation analysis and comparison to explore the influencing factors of overall survival (OS). Multivariate Cox regression was used to explore whether a factor was an independent influencing factor of OS.

### Identification of differentially expressed genes

Using the "limma" package for differential analysis of the data, genes with an adj-P value < 0.05 and a |log2-fold change|(FC)|> 1 were selected as differentially expressed genes. Among these genes, those with log2FC > 1 were considered upregulated genes, while genes with log2FC < -1 were considered downregulated genes. The differentially expressed genes were visualized using volcano plots. A Venn diagram was used to visualize the intersection of DEGs among the cohorts.

### Functional enrichment analysis

The genes were mapped to the background gene set, and gene ontology (GO) annotation was performed on the genes. The minimum gene set was set as 5, the maximum gene set was 5000, and 1000 resamplings were performed.

Gene set variation analysis (GSVA) is a specialized type of gene set enrichment method that transforms the expression matrix of genes across different samples into an expression matrix of gene sets across samples. This allows the assessment of whether different pathways are enriched across different samples. The GSVA R package (version 1.32.0) with default parameters was applied to evaluate the gene set enrichment scores of the two groups of samples [16]. The Wilcoxon rank-sum test was performed on the enrichment scores between the two groups to calculate the differential activity of gene sets between different groups.

Single sample gene set enrichment analysis (ssGSEA) ranked and normalized the gene expression values of a given sample and then calculated enrichment scores using the empirical cumulative distribution function of the genes in the signature and the remaining genes. Referring to the immune cell signature gene sets proposed by the team led by Charoentong, P., the GSVA package was utilized to quantitatively study the infiltration levels of 28 different immune cell types in the tumor microenvironment (TME) for each sample. The relative infiltration levels of each immune cell type are represented by enrichment scores from ssGSEA and were normalized to a uniform distribution ranging from 0 to 1.

The samples were divided into two groups by gene set enrichment analysis (GSEA). First, the differential fold values of all genes were ranked from large to small to represent the trend of gene expression between the two groups. After sorting, the top of the gene list can be seen as upregulated DEGs, while the bottom represents downregulated DEGs. Therefore, if the top of the target gene set is enriched, it can be said that overall, this gene set shows an upregulated trend. Conversely, if the enrichment is at the bottom, it indicates a decreasing trend. We obtained GSEA software (version 3.0) from the GSEA website (http://software.broadinstitute.org/gsea/index.jsp). The gene expression levels of the samples in the high- and low-expression groups were downloaded from the hallmark gene set to evaluate the relevant pathways and molecular mechanisms. Based on the gene expression profile and phenotype grouping, the minimum gene set was set as 5, the maximum gene set was set as 5000, and 1000 resamplings were used to select P < 0.05 and FDR < 0.05 as significantly enriched gene sets.

### scRNA-seq analysis of lung adenocarcinoma tissue

The GSE164789 cohort included 10X single-cell RNA sequencing data and contained a total of 79078 cells from 15 invasive lung adenocarcinoma samples. The Seurat 4.0 R package was used to extract mitochondrial-related genes from the 10X single-cell RNA sequencing data, filter out low-quality cells, and normalize the data. Principal component analysis (PCA) was performed on the hypervariable genes to reduce the dimensionality of the data. Finally, cell cycle analysis and normalization were performed, and the cell cycle fraction of each cell was calculated to exclude the influence of cell cycle-related genes.

The Harmony R package was used to remove batch effects from single-cell RNA sequencing data, and the DBSCAN method was applied to perform cell clustering. The three filtering criteria were as follows: a. cell clusters with fewer than 10 cells were removed, and clustering was performed again; b. cell populations with fewer than 20 cells were removed; and c. if a certain cell population was present in fewer than four samples, this cell population was removed. Uniform prevalence approximation and projection (UMAP) clustering of 10X single-cell RNA sequencing data was performed using the Seurat 4.0r package. The resolution was set to 0.25. Manually annotated cell names for the cell clusters obtained from UMAP clustering. The cell proportion of each cell population was calculated after cell annotation. Cell type-specific marker data were obtained from a single-cell study of lung cancer by Laughney and Bischoff et al. [17, 18].

The monocle2 package was used to transform the Seurat object into the CDS object of Monocle, calculate the size factor and dispersion, filter low-quality cells, perform semisupervised dimensionality reduction clustering, find the hypervariable genes needed for downstream pseudotime analysis, calculate the cell pseudotime, and display the cell time trajectory. The time sequence changes of the target genes in the cells were calculated.

### Statistical analysis

The statistical analysis in this study was conducted using R 4.3.2. For quantitative data that followed a normal distribution, a t test was performed, while for data that did not follow a normal distribution, a Wilcoxon test was used. When more than two groups were analysed, the Kruskal‒Wallis test was applied for nonparametric tests, while ANOVA was applied for parametric tests [19]. The calculation of the incidence rate of events was conducted using Fisher's exact test. The survival package was used to analyse the difference in prognosis between the two groups. The log-rank test was used to assess the significance of the differences in prognosis between different groups of patients. P < 0.05 (bilateral) was considered to indicate a statistically significant difference (ns, no statistically significant difference; *P < 0.05; **P < 0.01; ***P < 0.001; ****P < 0.0001). The false discovery rate (FDR) was tested by the Benjamini–Hochberg method [20].

## Results

### T-cell exclusion induced by MDSCs is the main immune escape mechanism affecting the survival of patients with lung adenocarcinoma

There are two different mechanisms of tumor immune evasion. One is the dysfunction of T cells in the TME. Second, the presence of immunosuppressive factors in the TME, such as MDSCs, M2 macrophages, and CAFs, prevents T-cell tumor infiltration. Exclusion and dysfunction scores and MDSC, M2, and CAF infiltration levels were calculated for each lung adenocarcinoma patient using the TIDE framework, as detailed in Table S1. The associations of exclusion, dysfunction, MDSCs, M2 macrophages, and CAFs with survival time and survival status were evaluated to identify the major immune escape mechanisms that threaten the survival of patients with lung adenocarcinoma [8]. Univariate Cox regression analysis revealed that only exclusion and MDSCs were risk factors for survival in all 5 lung adenocarcinoma cohorts (GSE41271, TCGA, GSE42127, GSE30219, and GSE14814), with P < 0.05 (Figure S1A-F).

The above analysis suggested that the immune escape mechanism of MDSCs by T cells has a significant impact on the survival of patients with lung adenocarcinoma. Based on the interquartile range, we simulated high, medium, and low infiltration levels of MDSCs in the lung adenocarcinoma TME and divided the samples into high, medium, and low infiltration groups. Survival analysis of the GSE41271 cohort revealed that the survival rate of patients in the high infiltration group was significantly lower than that in the low infiltration group (HR = 2.96, 95% CI 1.45, 6.03; P = 0.002) (Fig. 1A). Survival analysis of the TCGA cohort also revealed that the survival rate of patients in the high infiltration group was significantly lower than that in the low infiltration group (HR = 2.46, 95% CI 1.47, 4.10; P < 0.001) (Fig. 1B). The same analysis of the GSE42127, GSE30219, and GSE14814 cohorts revealed that the survival rate of patients with high MDSC infiltration was significantly lower (P < 0.05) (Figure S2A-C). In the GSE41271 cohort, multivariate Cox regression analysis revealed that high infiltration of MDSCs, an advanced pathological stage, was an independent prognostic factor for lung adenocarcinoma (HR = 2.52, 95% CI (1.17, 5.43), P < 0.05) (Fig. 1C). In the TCGA cohort, high infiltration of MDSCs, an advanced pathological stage, was an independent prognostic factor for lung adenocarcinoma (HR = 2.40, 95% CI (1.39, 4.41), P < 0.05) (Fig. 1D). In the GSE30219 and GSE14814 cohorts, high infiltration of MDSCs was an independent prognostic factor for LUAD (P < 0.05) (Figure S2E and F). There was no significant difference in the GSE42127 cohort (Figure S2D).

### MDSCs inhibit the antitumour immune response and promote tumor immune escape in lung adenocarcinoma

**Fig. 2:**
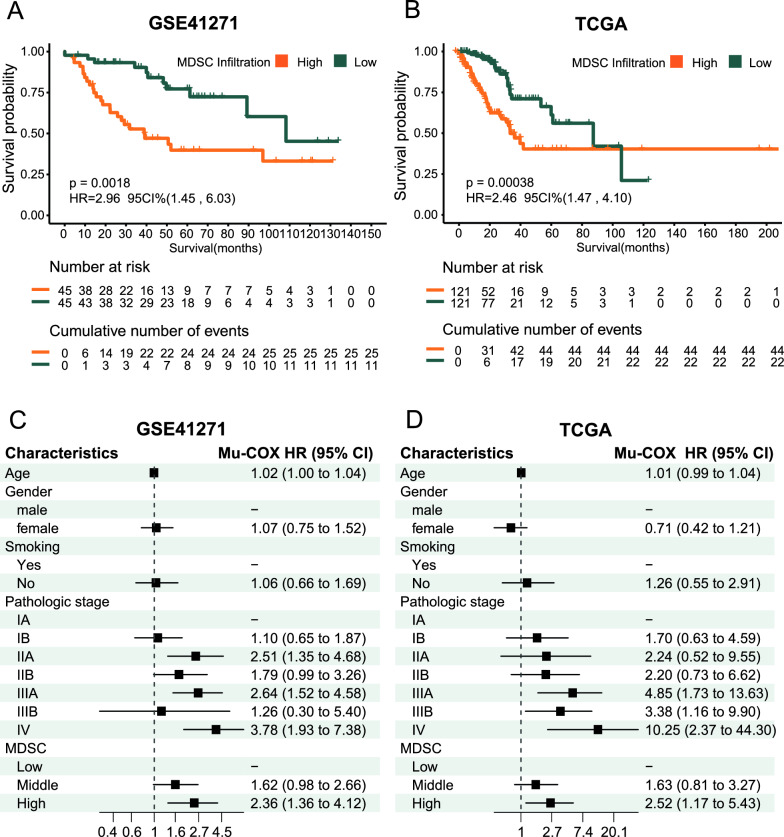
Survival analysis between groups with high and low MDSC infiltration in lung adenocarcinoma; **A** and **B** Kaplan‒Meier survival curves between the high- and low-invasive groups in the GSE41271 and TCGA cohorts; the log-rank test was used to test the significance of survival rates between groups. **C** and **D** Multivariate Cox regression analysis of the GSE41271 and TCGA lung adenocarcinoma cohorts. Mu-Cox multivariate Cox regression analysis, HR:, hazard ratio, 95% CI, 95% confidence interval

MDSCs play an important role in the immune escape of lung adenocarcinoma tumors, and their high infiltration level is an independent prognostic risk factor for lung adenocarcinoma patients. Patients in the 5 LUAD cohorts were divided into MDSC high- and low-infiltration groups according to the upper and lower quartiles, respectively. Differences were detected between the groups with high and low MDSC infiltration, and the results for the 5 cohorts are shown in Fig. 2A. A total of 373 common differentially expressed genes (Co-DEGs), including 132 upregulated genes and 241 downregulated genes (Fig. 2B and Table S2), were identified in the five cohorts. GO enrichment analysis revealed that the upregulated genes were enriched in biological processes such as the cell cycle, cell division, and mitosis. In addition, these genes were related to nucleotide metabolism and energy metabolism (Fig. 2C), and the downregulated genes were involved mainly in biological processes such as the immune response, immune activation, and immune regulation (Fig. 2E). KEGG enrichment analysis revealed that the upregulated genes were mainly enriched in DNA replication, mismatch repair, antigen processing and presentation, and glycolysis/gluconeogenesis (Fig. 2D), and the downregulated genes were mainly related to DNA replication, mismatch repair, antigen processing and presentation, and Th1 and Th2 cell differentiation (Fig. 2F). In conclusion, genes involved in cell proliferation-related processes such as mitosis, cell division, energy metabolism, and glycolysis were activated, while genes involved in the immune response, positive immune regulation, and immune activation were downregulated in the tumor microenvironment of lung adenocarcinoma patients with high MDSC infiltration.

**Fig. 3:**
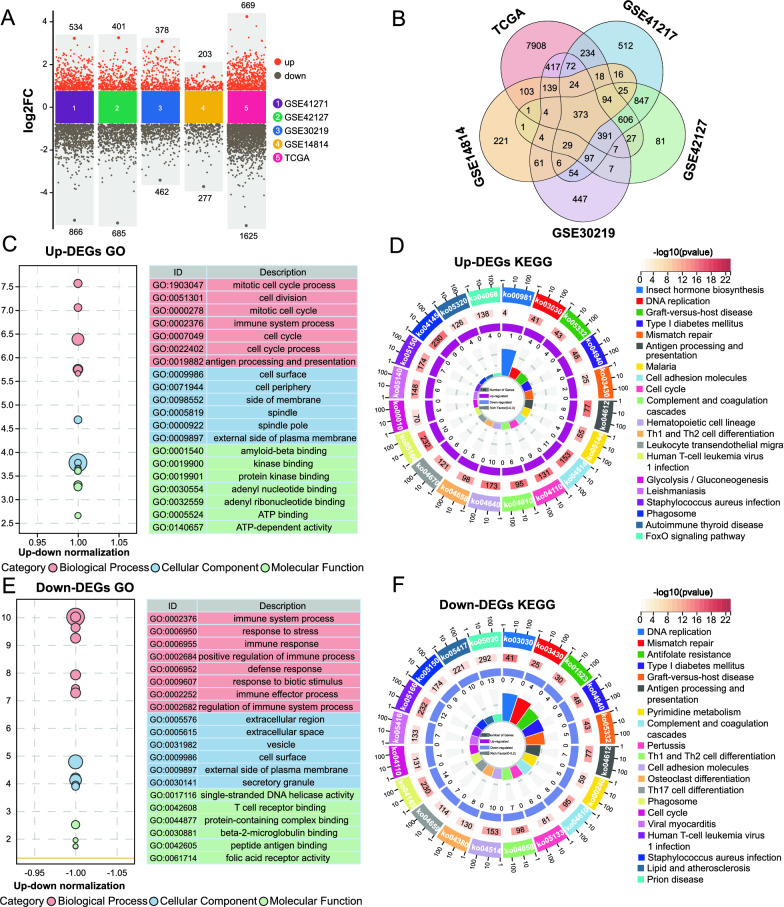
Functional enrichment analysis of DEGs between the MDSC-high and MDSC-low groups; **A** Volcano plot of DEGs in five LUAD cohorts. **B** Venn diagram of common DEGs in the five cohorts. **C** Bubble plot of upregulated DEGs according to Gene Ontology (GO) functional enrichment analysis, and the size of the circle represents the number of enriched genes. **D** KEGG enrichment analysis circle diagram of upregulated genes; KEGG: Kyoto Encyclopedia of Genes and Genomes. There are four circles from the outside to the inside. The first circle shows the enrichment classification and the coordinate ruler of the gene number. Different colors represent different classifications; 2. The second circle is the number of categories in the background genes and the P value. The more genes there are, the longer the bar. 3. The third circle shows the proportions of upregulated and downregulated genes in the bar graph; purple represents the proportion of upregulated genes, and blue represents the proportion of downregulated genes. The specific values are shown below. 4. The fourth circle is the RichFactor value of each category (the number of foreground genes divided by the number of background genes in the category), and a small cell represents 0.1. **E** Bubble plot of downregulated DEGs from the GO functional enrichment analysis. **F** KEGG enrichment analysis circle diagram of downregulated DEGs

MDSCs reportedly limit the infiltration of T cells and related antitumour immune cells in the TME [7]. The level of immune cell infiltration in the 5 lung adenocarcinoma cohorts was evaluated by the ssGSEA algorithm. In the GSE41271 cohort, there was a significant negative correlation between the infiltration level of MDSCs and each immune cell subtype (Fig. 3A). In addition, except for activated CD4 T cells, effector memory CD4 T cells, type 2 helper T cells, and dim CD56 natural killer cells, the infiltration levels of other immune cell subtypes were significantly reduced in the MDSC high infiltration group (P < 0.05) (Fig. 3D). The biological behavior of tumor cells (tumor proliferation rate, hypoxia, glycolysis, and epithelial–mesenchymal transition) and antitumour immune responses (antigen-presenting cell infiltration, antigen presentation, and T-cell toxicity) were specifically evaluated by the GSVA algorithm. In the GSE41271 cohort, the TME with high MDSC infiltration showed a greater tumor cell proliferation rate, severe hypoxia, and a greater level of glycolysis (Fig. 3B). Under the influence of several factors, epithelial cells lose their polarity and tight adhesion between cells, gain infiltration and migration abilities and transform into cells with interstitial cell characteristics. This behavior enables cells to undergo transfer and infiltration, thereby accelerating immune escape [21]. In the GSE41271 cohort, epithelial–mesenchymal transition (EMT signature) was more pronounced in the TME with high MDSC infiltration (Fig. 3B). In the GSE41271 cohort, the levels of macrophages and DC trafficking and antigen presentation ability in the TME with high MDSC infiltration were significantly reduced. In addition, T-cell cytotoxicity was also significantly reduced (Fig. 3C). Similar results were obtained from the same analysis described above for the TCGA, GSE42127, GSE30219, and GSE14814 cohorts (Figure S3-S6).

In conclusion, high MDSC infiltration in the TME of lung adenocarcinoma significantly inhibited the antitumour immune response but enhanced the immune escape ability of tumor cells.

### IRF8 deficiency induces the differentiation and formation of MDSCs in lung adenocarcinoma

A high infiltration level of MDSCs accelerates tumor immune escape in lung adenocarcinoma. Moreover, high levels of MDSCs, an advanced pathological stage, were found to be independent prognostic factors for lung adenocarcinoma. Therefore, it is highly important to explore the upstream target molecules that induce MDSC formation.

**Fig. 4:**
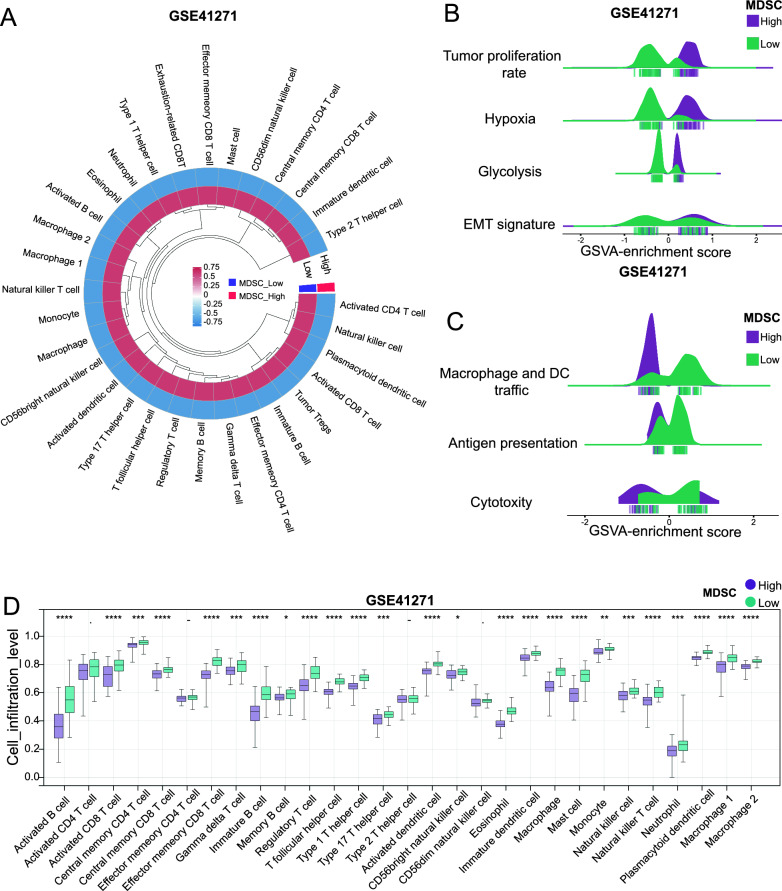
Assessment of antitumour immune responses between MDSCs with high and low infiltration in the GSE41271 cohort; **A** Correlation heatmap of circular lines between MDSCs and other immune cell infiltration levels in lung adenocarcinoma. **B** Ridge plot of differences in the biological behavior of tumor cells between MDSCs with high and low infiltration. **C** Ridge plot of the differences in antitumour immune responses between MDSCs with high and low infiltration. **D** Box plot of the differences in the infiltration levels of immune cells between MDSCs with high and low infiltration. −, no significant difference; *P < 0.05; **P < 0.01; ***P < 0.001; ****P < 0.0001

To explore the factors affecting the infiltration of MDSCs, we conducted a correlation analysis between a total of 373 Co-DEGs and the infiltration level of MDSCs in 5 lung adenocarcinoma cohorts while retaining Co-DEGs with a | R |≥ 0.55. A total of 45 co-DEGs were associated with the MDSC infiltration level (| R | acuity 0.55, P < 0.05) (Fig. 4A). Figure 4B shows the 45 genes, among which IRF8, EVI2B, CD37, and DPEP2 had the strongest correlation, with R ≤ −0.70 and P < 0.05. Previous studies have demonstrated that impaired differentiation of myeloid cells leads to their differentiation into MDSCs [22, 23]. GO enrichment analysis of the C5 gene set was performed on 45 highly correlated co-DEGs to screen DEGs related to myeloid cell differentiation. C5-GO enrichment analysis revealed that IRF8, CD4, RASSF2, and EVI2B were involved in the development and differentiation of myeloid cells such as macrophages, dendritic cells, and monocytes. Differential analysis between tumor and adjacent lung adenocarcinoma tissue samples from the TCGA and GETx databases was performed. The results showed that IRF8, RASSF2, and EVI2B were significantly downregulated in lung adenocarcinoma tumors. The expression of CD4 was not significantly different. Therefore, we hypothesized that the downregulation of IRF8, CD4, RASSF2, and EVI2B may impair myeloid differentiation and induce myeloid differentiation toward MDSCs.

**Fig. 5:**
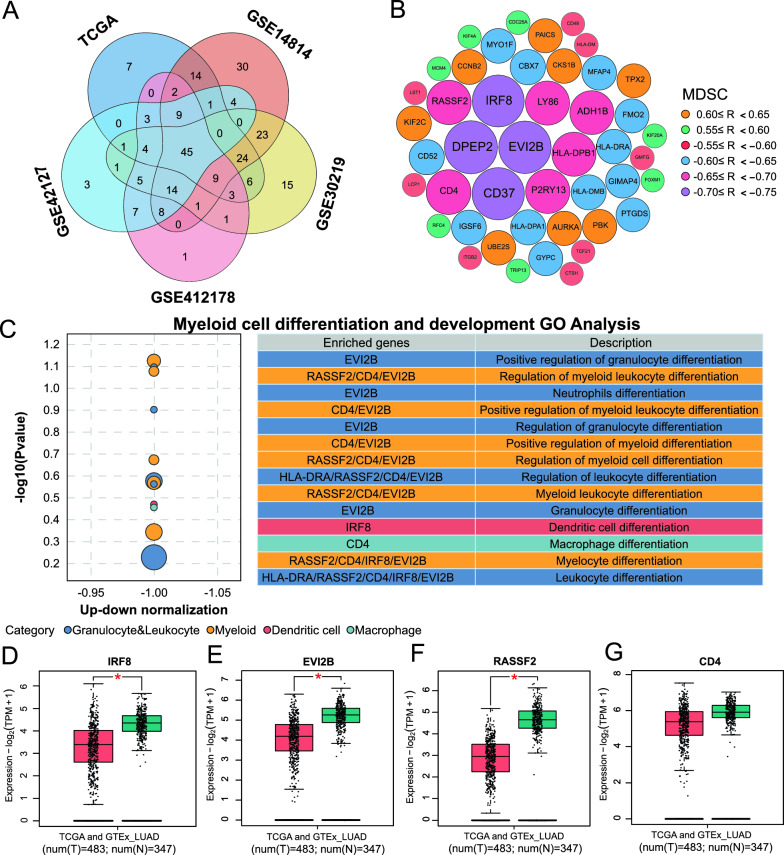
Identification of genes involved in the differentiation of MDSCs. **A** Venn diagram of DEGs strongly associated with MDSC infiltration in all 5 cohorts. **B** Bubble map of genes strongly related to MDSC infiltration (a total of 45 genes); the size of the bubble represents the correlation. **C** Bubble plots of enrichment analysis to identify genes that affect myeloid cell differentiation and development; **D**–**G** Box plots of differences in IRF8, EVI2B, RASSF2, and CD4 expression between LUAD tissues and normal tissues; T tumor tissue, N normal tissue

Using the GSE164789 single-cell RNA-seq cohort, we investigated the variations in the expression of IRF8, CD4, RASSF2, and EVI2B during myeloid differentiation. UMAP analysis and visualization revealed various cell types in the lung adenocarcinoma TME. Among these cells, myeloid cell populations, including macrophages, myeloid dendritic cells, monocytes, and MDSCs, were identified (Fig. 5A). All cell types and cell markers are shown in Figure S7A and B. Figure 5B shows the proportion of each cell population in the TME. Localization analysis of genes of interest in cell subpopulations revealed that IRF8, CD4, RASSF2, and EVI2B were consistently highly expressed in myeloid cells (Figure S7C). We further investigated the expression of IRF8, CD4, RASSF2, and EVI2B in myeloid subtypes of lung adenocarcinoma. Figure 5C shows that IRF8 was highly expressed in macrophages, myeloid dendritic cells, and monocytes but was significantly downregulated in MDSCs. CD4 and EVI2B were stably highly expressed in all four types of myeloid cells. RASSF2 was highly expressed in myeloid dendritic cells and monocytes and weakly expressed in macrophages and MDSCs.

Single-cell pseudotemporal analysis was performed on myeloid cell populations as well as on various myeloid cell subtypes. Figure 5D–E show the developmental and state trajectories of myeloid cells; since we focused on the variation of genes of interest with cell development, we did not analyse further at the branch point. Figure 5F shows the time trajectory of cell development, from which it can be seen that the myeloid cells gradually matured from left to right. Figure 5G–H show that IRF8 was upregulated at the later stage of the myeloid cell progression. However, CD4, RASSF2, and EVI2B did not change significantly with the duration of myeloid cell culture (Fig. 7D–E). Figure 5I, J show the developmental and state trajectories of MDSCs. Figure 5K–M shows the relationship between the developmental time trajectory of MDSCs and the expression of IRF8. In the early stages of MDSC development, IRF8 expression is high, and over time, IRF8 expression rapidly decreases and remains at a low level for a long time. However, CD4, RASSF2, and EVI2B did not change significantly with the development of MDSCs (Figure S7F and G). Figure 5N, O show the developmental and state trajectories of myeloid dendritic cells. Figure 5P–R shows that IRF8 expression gradually increased over time during myeloid dendritic cell development. With the development of myeloid dendritic cells, EVI2B expression gradually decreased, CD4 expression gradually increased, and RASSF2 expression did not significantly change (Figure S7 H and I). Figure S8A shows that CD4 expression first increased and then decreased with the development of macrophages, while IRF8, RASSF2, and EVI2B expression did not significantly change. Figure S8B shows that EVI2B expression first decreased and then gradually increased during monocyte development, while IRF8, RASSF2, and CD4 expression did not significantly change.

In summary, IRF8 is stably highly expressed in myeloid cells, whereas it is significantly downregulated in MDSCs of myeloid cell subtypes. Single-cell pseudotemporal analysis revealed that IRF8 expression was high in the early stages of MDSC development but rapidly decreased with cell development and maintained a low expression state for a long time. Therefore, these results confirmed our hypothesis that the downregulation of IRF8 impaired the normal development of myeloid cells and induced the differentiation and development of myeloid cells toward MDSCs.

### IRF8 induces the differentiation and maturation of MDSCs by regulating the IL6-JAK-STAT3 pathway

**Fig. 6:**
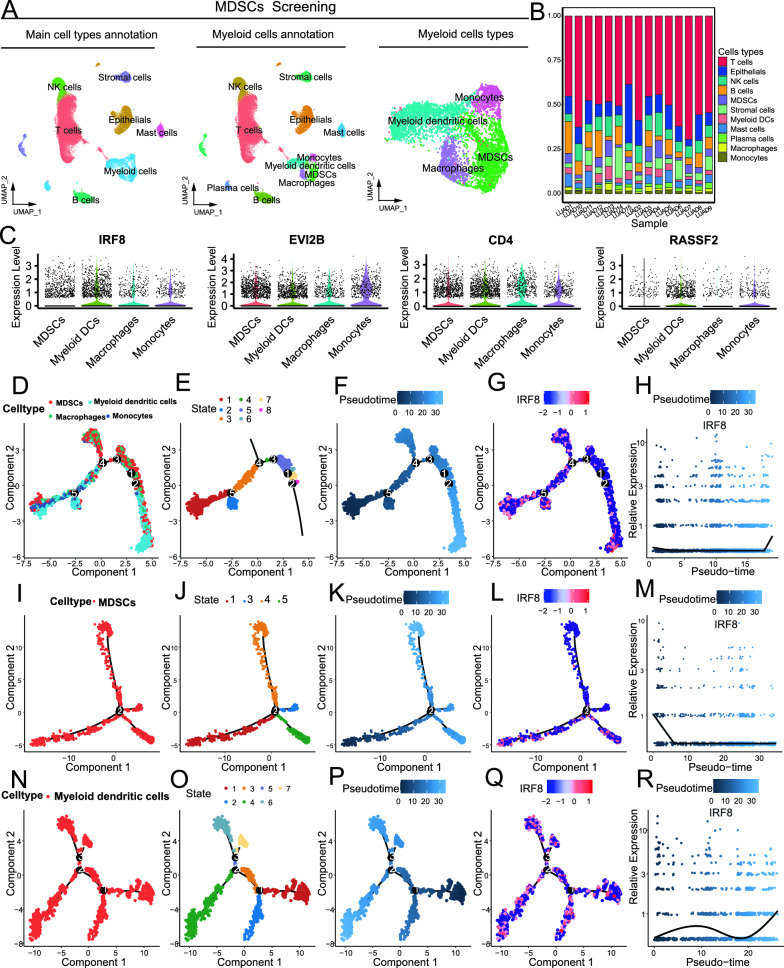
Single-cell RNA-Seq Analysis of Lung Adenocarcinoma; **A** UMAP of cell clustering and annotation. **B** Proportional plot of the distribution of different cells in the TME of lung adenocarcinoma. **C** Violin plot of IRF8, EVI2B, RASSF2, and CD4 expression in myeloid cell subsets. **D** Developmental trajectories of myeloid cell subsets. **E** Trajectories of developmental states of myeloid cell subsets. **F** Temporal trajectories of myeloid cell subset development. **G** Trajectories of IRF8 distribution in myeloid cell subsets. **H** Map of IRF8 distribution in myeloid cell subsets. **I** Developmental trajectories of MDSCs. **J** Developmental state trajectories of MDSCs. **K** Time trajectory of MDSC development. **L** IRF8 distribution trajectories in MDSC populations. **M** IRF8 distribution map in MDSC populations. **N** Myeloid dendritic cell developmental trajectories. **O** Trajectories of the developmental states of myeloid dendritic cells. **P** Time trajectories of myeloid dendritic cell development. **Q** IRF8 distribution trajectories in myeloid dendritic cell populations. **R** IRF8 distribution map in myeloid dendritic cell populations

Studies have confirmed that STAT3 is involved in all stages of MDSC development [24]. Research on breast tumor-bearing mice also confirmed that the IRF8-STAT3 axis regulates the development of MDSCs [25]. The 5 lung adenocarcinoma cohorts were divided into high- and low-MDSC infiltration groups and high- and low-IRF8 expression groups, respectively, with the upper and lower quartiles as the cut-off points. GSEA was applied to compare hallmark characteristics between groups with high and low MDSC infiltration and between groups with high and low IRF8 expression, and the top 5 and/or 6 results are presented. In both the GSE41271 and TCGA lung adenocarcinoma cohorts, the IL6-JAK-STAT3 pathway was activated in the MDSC high infiltration group, while the IL6-JAK-STAT3 pathway was inhibited in the IRF8 high-expression group (Fig. 6A, B). The results were similar in the GSE42127, GSE30219, and GSE14814 cohorts (Figure S9A-C).

Based on the GSE164789 lung adenocarcinoma single-cell RNA-seq cohort, the variations in the expression of STAT3 and JAK1, the core genes in the IL6-JAK-STAT3 pathway, were calculated based on the developmental trajectory of MDSCs. The results showed that STAT3 and JAK1 were rapidly upregulated during the late developmental stages of MDSCs (Fig. 6C, D). Therefore, we speculate that IRF8 may affect MDSC differentiation by regulating the IL6-JAK-STAT3 pathway in lung adenocarcinoma. IRF8 is an independent prognostic factor for lung adenocarcinoma.

### IRF8 is an independent prognostic factor for lung adenocarcinoma

**Fig. 7:**
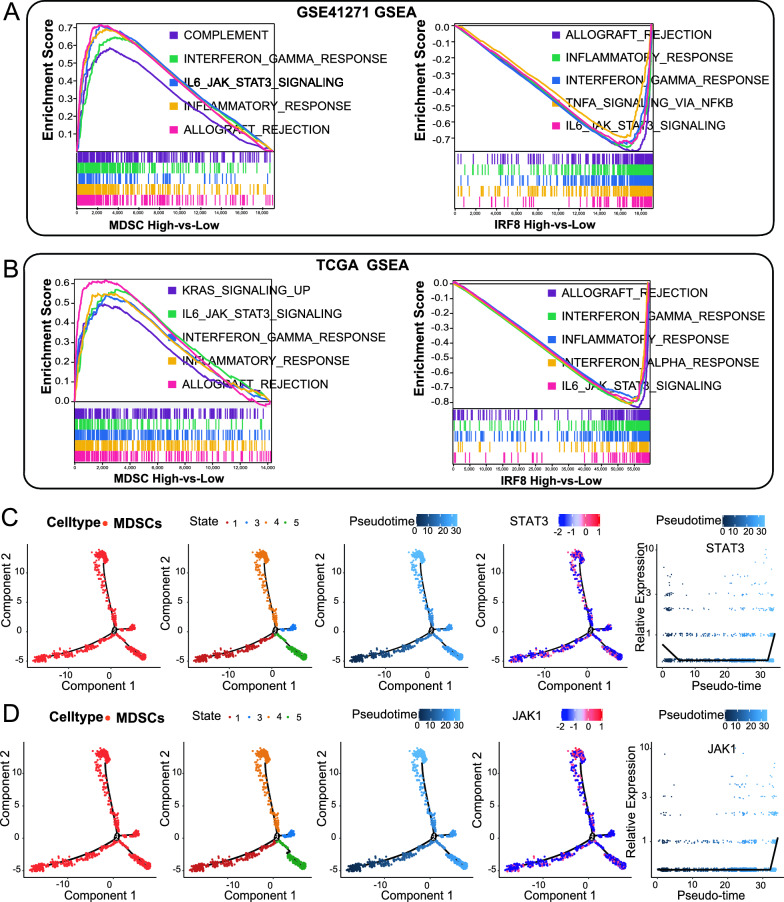
Identification of signalling pathways regulating the differentiation of MDSCs **A** GSEA between groups with high and low MDSC infiltration and groups with high and low IRF8 expression in the GSE41271 cohort; **B** GSEA between groups with high and low MDSC infiltration and groups with high and low IRF8 expression in the TCGA cohort; **C** Plot of STAT3 distribution in the developmental trajectory of MDSC populations; **D** Plot of JAK1 distribution in the developmental trajectory of MDSC populations

The expression of IRF8 is closely related to the development of MDSCs in the tumor microenvironment of lung adenocarcinoma. Therefore, we explored the practical clinical significance of variations in the expression of IRF8 in lung adenocarcinoma patients. The 5 lung adenocarcinoma cohorts were, respectively simulated as high and low IRF8 expression groups with the upper and lower quartiles as the cut-off points. The KM curve of the GSE41217 cohort showed that IRF8 was a protective factor for survival (HR = 0.35, 95% CI 0.17, 0.72; P < 0.05) (Fig. 7A). The KM curve of the GSE42127 cohort showed that IRF8 was a protective factor for survival (HR = 0.35, 0.13, 0.96; P < 0.05) (Fig. 7B). The KM curve of the TCGA cohort showed that IRF8 was a protective factor for survival (HR = 0.54, 0.33, 0.88; P < 0.05) (Fig. 7C). In the GSE30219 and GSE14814 cohorts, the expression of IRF8 had no significant impact on survival. In the GSE41271, GSE42127, and TCGA cohorts, multivariate Cox analysis confirmed that high expression of IRF8 is a protective independent prognostic factor for lung adenocarcinoma (Fig. 7D–F).

## Discussion

**Fig. 8:**
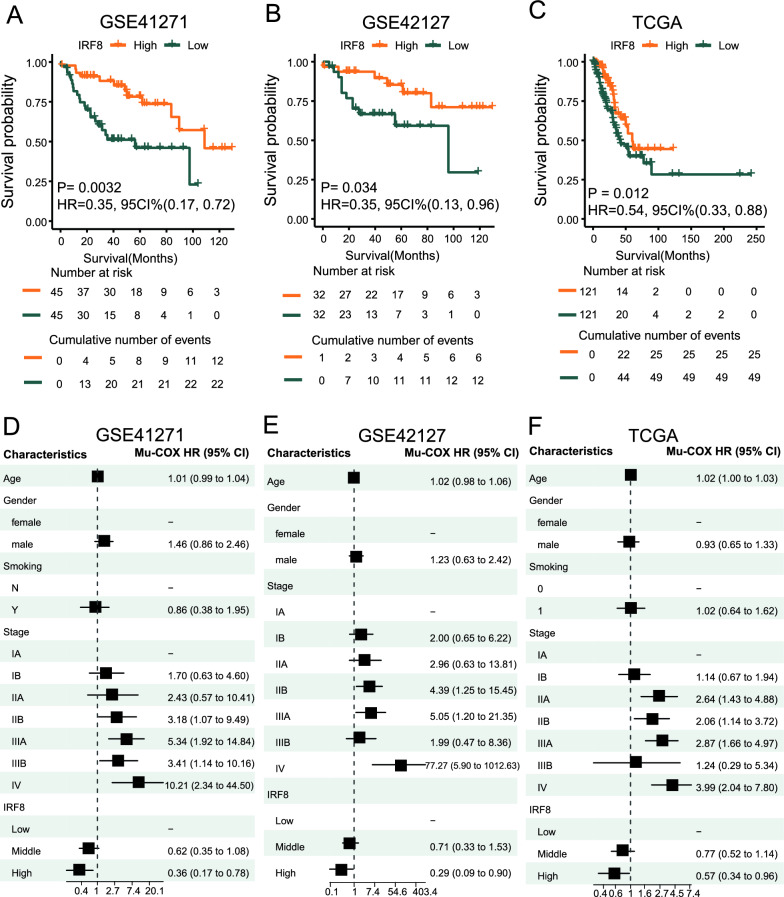
Survival analysis between lung adenocarcinoma patients with high and low IRF8 expression. **A**–**C** Kaplan‒Meier survival curves between the high- and low-expression groups in the GSE41271, GSE42127, and TCGA cohorts; a log-rank test was used to test the significance of differences in survival rates between groups. **C** and **D**: Multivariate Cox regression analysis between the high and low-expression groups in the GSE41271, GSE42127, and TCGA cohorts; Mu-Cox multivariate Cox regression analysis, HR hazard ratio 95% CI 95% confidence interval

There are two different immune mechanisms of tumor immune escape in the TME: T-cell dysfunction and exclusion [6, 7]. In this study, it was found that only exclusion, an immune escape mechanism, had a significant impact on the survival prognosis of patients with lung adenocarcinoma. CAFs, MDSCs, and M2 macrophages are the main cell types that restrict T-cell entry into tumor tissues. Survival analysis of 954 patients in 5 lung adenocarcinoma cohorts revealed that only the infiltration level of MDSCs was a serious threat to survival prognosis, and a high infiltration level of MDSCs, similar to advanced tumor stage, was an independent prognostic factor. In addition, we found that the high infiltration of MDSCs in lung adenocarcinoma significantly inhibited the antitumour immune response and promoted tumor proliferation and tumor cell mesenchymal transition. This suggests that the tumor microenvironment with high infiltration levels of MDSCs is devoid of immune surveillance and that tumor cells grow aggressively and undergo distant metastasis. The accumulation of MDSCs in the TME is the main immune escape mechanism in lung adenocarcinoma. Thus, exploring the infiltration mechanism of MDSCs is highly important for immunotherapy for lung adenocarcinoma (Fig. 8).

MDSCs consist of a mixture of myeloid-derived cells at different stages of differentiation, and their phenotypes are complex and vary with tumor type [26, 27]. This study suggested that MDSCs play an important role in tumor immune escape in lung adenocarcinoma, but few studies have reported the pathogenesis and progression of MDSCs in lung adenocarcinoma. A study based on breast tumor-bearing mice showed that the IRF8-STAT3 axis regulates the development of MDSCs and that IRF8 deficiency is positively correlated with the accumulation of MDSCs in tumors [25]. IRF8 is a complete transcription factor involved in the differentiation and lineage commitment of myeloid cells and participates in different stages of myelopoiesis [28–31]. Our study showed that IRF8 expression was high in the early stages of MDSC development but rapidly decreased with cell development and maintained a low expression state for a long time in lung adenocarcinoma. In summary, IRF8 deficiency may impair the normal development of myeloid cells and induce the differentiation and development of myeloid cells toward MDSCs. In addition, we found that IRF8 was significantly upregulated during dendritic cell development and maturation. Recent studies have also demonstrated that high expression of IRF8 is required for the formation of type 1 classical dendritic cells (cDC1s) [32]. Human cDC1s perform excellently in antitumour cellular immunity. cDC1s migrate to tumor-draining lymph nodes to activate CD8 + T cells, present tumor antigens, recruit CD8 + T cells, and secrete cytokines in the TME, thereby enhancing the local tumor immune response [33]. Therefore, activating IRF8 may be another interesting targeted approach for the immunotherapy of lung adenocarcinoma.

In a recent review describing MDSCs, the molecular pathways regulating the development of MDSCs, which mainly include factors such as IRF8, STAT3, STAT1, and STAT6, were identified [24]. Our single-cell pseudotemporal analysis showed that STAT3 and JAK1 were significantly upregulated during MDSC development, which was consistent with the activation of the IL6-JAK-STAT3 pathway by the increased infiltration of MDSCs. Jeremy D. et al. reported that IRF8 deletion activates the STAT3 pathway to promote the accumulation of MDSCs in mice with breast cancer [25]. In addition, we found a significant inverse correlation between IRF8 expression and MDSC infiltration in the lung adenocarcinoma TME. Therefore, we inferred that downregulation of IRF8 might induce MDSC formation by activating the IL6-JAK-STAT3 pathway in lung adenocarcinoma.

MDSCs have a short survival time in the TME, and they achieve long-lasting immunosuppression by continuous recruitment to the target area. Because of their short lifespan, it is difficult to reverse their activation state. Therefore, blocking the differentiation of MDSCs, inhibiting their infiltration into tumor tissues, or directly targeting them may be effective treatments. Our work showed that a high infiltration level of MDSCs, similar to advanced tumor stage, was an independent prognostic factor. This conclusion was obtained from the analysis of the survival of 954 patients in 5 lung adenocarcinoma cohorts, and we believe that this conclusion is reliable. In a phase II clinical trial that directly targeted MDSCs, a monoclonal anti-CD33 antibody combined with a toxin was shown to deplete CD33-expressing MDSCs with promising efficacy [34–37]. These findings undoubtedly provide hope for the use of immunotherapy for lung adenocarcinoma patients. Hopefully, our single-cell pseudotemporal analysis showed that IRF8 was rapidly down-regulated and maintained at a long-term low level as MDSC developed and matured, and IRF8 was also down-regulated in lung adenocarcinomas. This may be because IRF8 deficiency impairs the normal differentiation of myeloid cells, promoting the formation and accumulation of MDSCs. In addition, our study showed that high expression of IRF8 was an independent protective factor for lung adenocarcinoma. Therefore, we suggest that targeting the activation of IRF8 to inhibit the differentiation of MDSCs may provide a new approach for immunizing lung adenocarcinoma.

The study still has limitations. IRF8 promotes MDSC formation in lung adenocarcinoma which lacks experimental verification, especially since IRF8 regulates MDSC differentiation through the IL6-JAK-STAT3 pathway, which only provides us with theoretical insights. Our subsequent studies will focus on immune escape animal models, cell co-culture, and functional detection by which IRF8 induces MDSC formation.

## Conclusion

IRF8 deficiency impaired the normal development of myeloid cells and induced the differentiation of myeloid cells toward MDSCs, thereby accelerating the immune escape of lung adenocarcinoma cells. Targeting the activation of IRF8 may be a new immunotherapy strategy for lung adenocarcinoma.

### Supplementary Information

Additional file 1 Figure S1. Identification of immune escape factors in lung adenocarcinoma patients. A-F: Forest plot of survival analysis of the main factors related to immune escape in the 5 lung adenocarcinoma cohorts. Exclusion: T-cell exclusion; CAFs: Cancer-associated fibroblasts, MDSCs: Myeloid-derived suppressor cells; M2: M2 subtype of tumor-associated macrophages; Dysfunction: T-cell dysfunction. F: Venn diagram showing the immune escape factors with a significant impact on survival in the 5 cohorts. Figure S2. Survival analysis between groups with high and low MDSC infiltration in lung adenocarcinoma. A-C: Kaplan‒Meier survival curves between the high- and low-invasive groups in the GSE42127, GSE30219, and GSE14814 cohorts; the log-rank test was used to test the significance of survival rates between groups. D-F: Multivariate Cox regression analysis of the GSE42127, GSE30219, and GSE14814 lung adenocarcinoma cohorts. Mu-Cox multivariate Cox regression analysis; HR: hazard ratio; 95% CI: 95% confidence interval. Figure S3. Assessment of antitumour immune responses between MDSCs with high and low infiltration in the TCGA cohort. A: Correlation heatmap of circular lines between MDSCs and other immune cell infiltration levels in lung adenocarcinoma. B: Ridge plot of differences in the biological behavior of tumor cells between MDSCs with high and low infiltration. C: Violin plot of the differences in antitumour immune responses between MDSCs with high and low infiltration. D: Box plot of the differences in the infiltration levels of immune cells between MDSCs with high and low infiltration. -, no significant difference; *, P < 0.05; **, P < 0.01; ***, P < 0.001; ****, P < 0.0001. Figure S4. Assessment of antitumour immune responses between MDSCs with high and low infiltration in the GSE42127 cohort; A: Correlation heatmap of circular lines between MDSCs and other immune cell infiltration levels in lung adenocarcinoma. B: Violin plot of differences in the biological behavior of tumor cells between MDSCs with high and low infiltration. C: Violin plot of the differences in antitumour immune responses between MDSCs with high and low infiltration. D: Box plot of the differences in the infiltration levels of immune cells between MDSCs with high and low infiltration. -, no significant difference; *, P < 0.05; **, P < 0.01; ***, P < 0.001; ****, P < 0.0001. Figure S5. Assessment of antitumour immune responses between MDSCs with high and low infiltration in the GSE30219 cohort; A: Correlation heatmap of circular lines between MDSCs and other immune cell infiltration levels in lung adenocarcinoma. B: Violin plot of differences in the biological behavior of tumor cells between MDSCs with high and low infiltration. C: Violin plot of the differences in antitumour immune responses between MDSCs with high and low infiltration. D: Box plot of the differences in the infiltration levels of immune cells between MDSCs with high and low infiltration. -, no significant difference; *, P < 0.05; **, P < 0.01; ***, P < 0.001; ****, P < 0.0001. Figure S6. Assessment of antitumour immune responses between MDSCs with high and low infiltration in the GSE14814 cohort; A: Correlation heatmap of circular lines between MDSCs and other immune cell infiltration levels in lung adenocarcinoma. B: Violin plot of differences in the biological behavior of tumor cells between MDSCs with high and low infiltration. C: Violin plot of the differences in antitumour immune responses between MDSCs with high and low infiltration. D: Box plot of the differences in the infiltration levels of immune cells between MDSCs with high and low infiltration. -, no significant difference; *, P < 0.05; **, P < 0.01; ***, P < 0.001; ****, P < 0.0001. Figure S7. Single-cell RNA-Seq analysis of lung adenocarcinoma; A: UMAP after data filtering and quality control. B: Mean expression and percentage expression of selected marker genes in cell types. C: Violin plot of IRF8, EVI2B, RASSF2, and CD4 expression in the main cell subsets. D: Trajectories of EVI2B, RASSF2, and CD4 distribution in myeloid cell subsets. E: Map of EVI2B, RASSF2, and CD4 distribution in myeloid cell subsets. F: Trajectories of EVI2B, RASSF2, and CD4 distribution in MDSC populations. G: Map of EVI2B, RASSF2, and CD4 distribution in MDSC populations. H: Distribution of EVI2B, RASSF2, and CD4 trajectories in myeloid dendritic cell populations. I: Map of EVI2B, RASSF2, and CD4 distribution in myeloid dendritic cell populations. Figure S8. Single-cell RNA-Seq Analysis of Lung Adenocarcinoma; A: Temporal trajectories of macrophages and distribution of IRF8, EVI2B, RASSF2, and CD4. B: Temporal trajectories of monocytes and distribution of IRF8, EVI2B, RASSF2, and CD4. Figure S9. Identification of signalling pathways regulating the differentiation of MDSCs A: GSEA between groups with high and low MDSC infiltration and groups with high and low IRF8 expression in the GSE42127 cohort; B: GSEA between groups with high and low MDSC infiltration and groups with high and low IRF8 expression in the GSE30219 cohort; C: GSEA between groups with high and low MDSC infiltration and groups with high and low IRF8 expression in the GSE14814 cohort.

Additional file 2.

Additional file 3.

## Data Availability

Publicly available datasets were analysed in this study. This data can be found below: the NCBI Gene Expression Omnibus database (https://www.ncbi.nlm.nih.gov/); TCGA database (https://www.cancer.gov/ccg/research/genome-sequencing/tcga); Concrete data and requests for materials should be addressed to Zhongmin Peng.

## Abbreviations

cDC1: CDC1
CAF: Cancer-associated fibroblasts
DBSCAN: Density-based spatial clustering of applications
DEGs: Differentially expressed genes
EMT: Epithelial–mesenchymal transition
FDR: False discovery rate
GEO: Tumor microenvironment
GO: Gene ontology
GSVA: Gene set variation analysis
GTEx: Genotype-tissue expression
IL-6: Interleukin 6
IRF8: Interferon regulatory factor 8
JAK1: Janus kinase1
KEGG: Kyoto encyclopedia of genes and genomes
LUAD: Lung adenocarcinoma
M2: M2 subtype of tumor-associated macrophages
MDSCs: Myeloid-derived suppressor cells
NK cells: Natural killer cells
OS: Overall survival
PD-1: Programmed death-1
PD-L1: Programmed death-ligand 1
PPI: Protein‒protein interaction
RNA: Ribonucleic acid
STAT3: Signal transducer and activator of transcription 3
ssGSEA: Single sample gene set enrichment analysis
TCGA: The cancer genome atlas
TIDE: Tumor immune dysfunction and exclusion
TME: Tumor microenvironment
TPM: Transcripts per million reads
Tregs: Regulatory T cells
UMAP: Uniform manifold approximation and projection
