# Supplementary material for: IRF8 deficiency-induced myeloid-derived suppressor cell promote immune evasion in lung adenocarcinoma
Source: J Transl Med. 2024 Jul 24;22:678. doi: 10.1186/s12967-024-05519-7 (PMC11270856; doi:10.1186/s12967-024-05519-7)
Supplement: Supplementary file 1 — Additional file 1 Figure S1. Identification of immune escape factors in lung adenocarcinoma patients. A-F: Forest plot of survival analysis of the main factors related to immune escape in the 5 lung adenocarcinoma cohorts. Exclusion: T-cell exclusion; CAFs: Cancer-associated fibroblasts, MDSCs: Myeloid-derived suppressor cells; M2: M2 subtype of tumor-associated macrophages; Dysfunction: T-cell dysfunction. F: Venn diagram showing the immune escape factors with a significant impact on survival in the 5 cohorts. Figure S2. Survival analysis between groups with high and low MDSC infiltration in lung adenocarcinoma. A-C: Kaplan‒Meier survival curves between the high- and low-invasive groups in the GSE42127, GSE30219, and GSE14814 cohorts; the log-rank test was used to test the significance of survival rates between groups. D-F: Multivariate Cox regression analysis of the GSE42127, GSE30219, and GSE14814 lung adenocarcinoma cohorts. Mu-Cox multivariate Cox regression analysis; HR: hazard ratio; 95% CI: 95% confidence interval. Figure S3. Assessment of antitumour immune responses between MDSCs with high and low infiltration in the TCGA cohort. A: Correlation heatmap of circular lines between MDSCs and other immune cell infiltration levels in lung adenocarcinoma. B: Ridge plot of differences in the biological behavior of tumor cells between MDSCs with high and low infiltration. C: Violin plot of the differences in antitumour immune responses between MDSCs with high and low infiltration. D: Box plot of the differences in the infiltration levels of immune cells between MDSCs with high and low infiltration. -, no significant difference; *, P < 0.05; **, P < 0.01; ***, P < 0.001; ****, P < 0.0001. Figure S4. Assessment of antitumour immune responses between MDSCs with high and low infiltration in the GSE42127 cohort; A: Correlation heatmap of circular lines between MDSCs and other immune cell infiltration levels in lung adenocarcinoma. B: Violin plot of differences in the bio [file 12967_2024_5519_MOESM1_ESM.docx]

**Supplementary Materials**

**Figure S1**

**
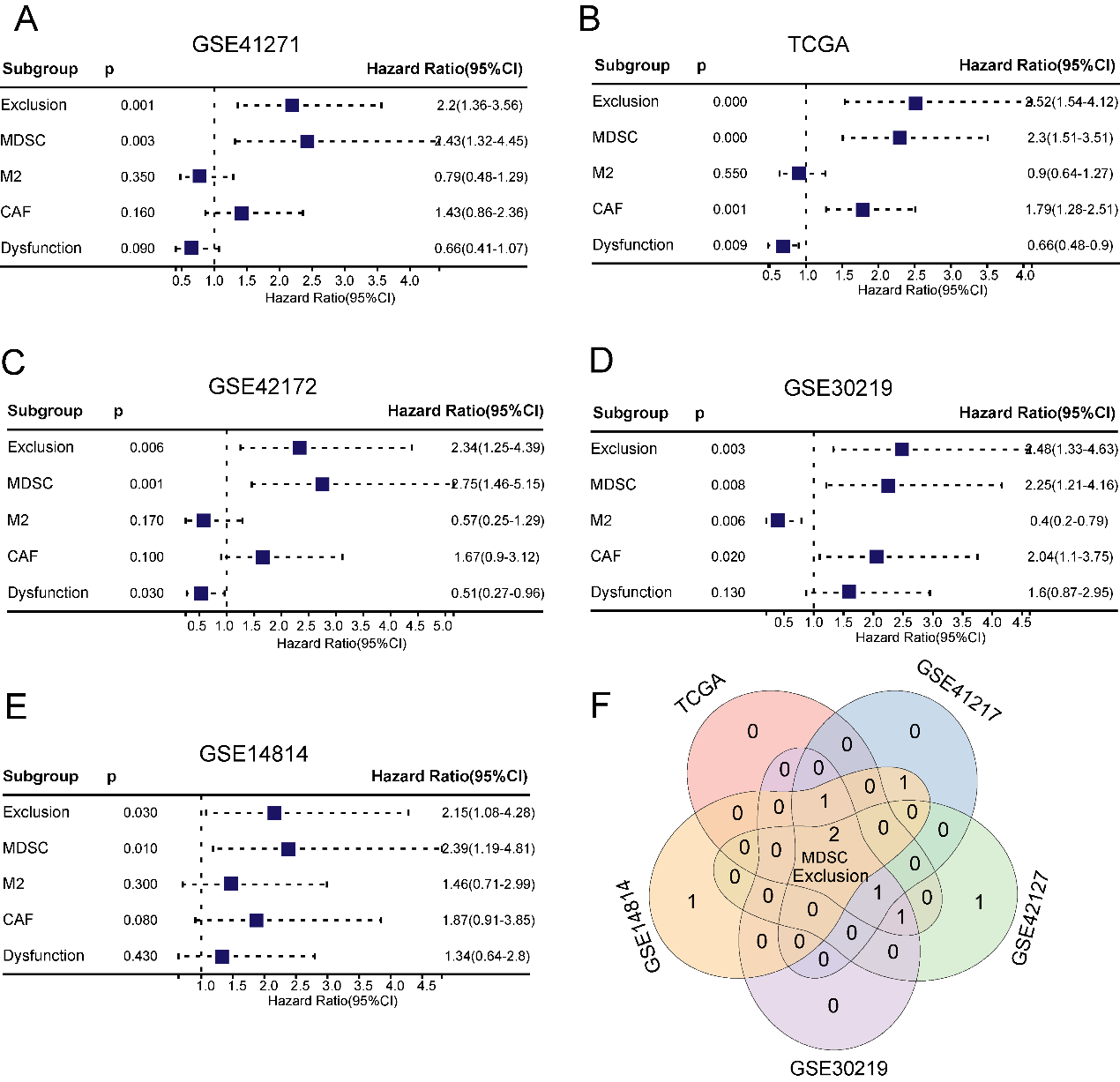
**

**Figure S1.** Identification of immune escape factors in lung adenocarcinoma patients. **A-F:** Forest plot of survival analysis of the main factors related to immune escape in the 5 lung adenocarcinoma cohorts. Exclusion: T-cell exclusion; CAFs: Cancer-associated fibroblasts, MDSCs: Myeloid-derived suppressor cells; M2: M2 subtype of tumor-associated macrophages; Dysfunction: T-cell dysfunction. **E:** Venn diagram showing the immune escape factors with a significant impact on survival in the 5 cohorts.

**Figure S2**

**
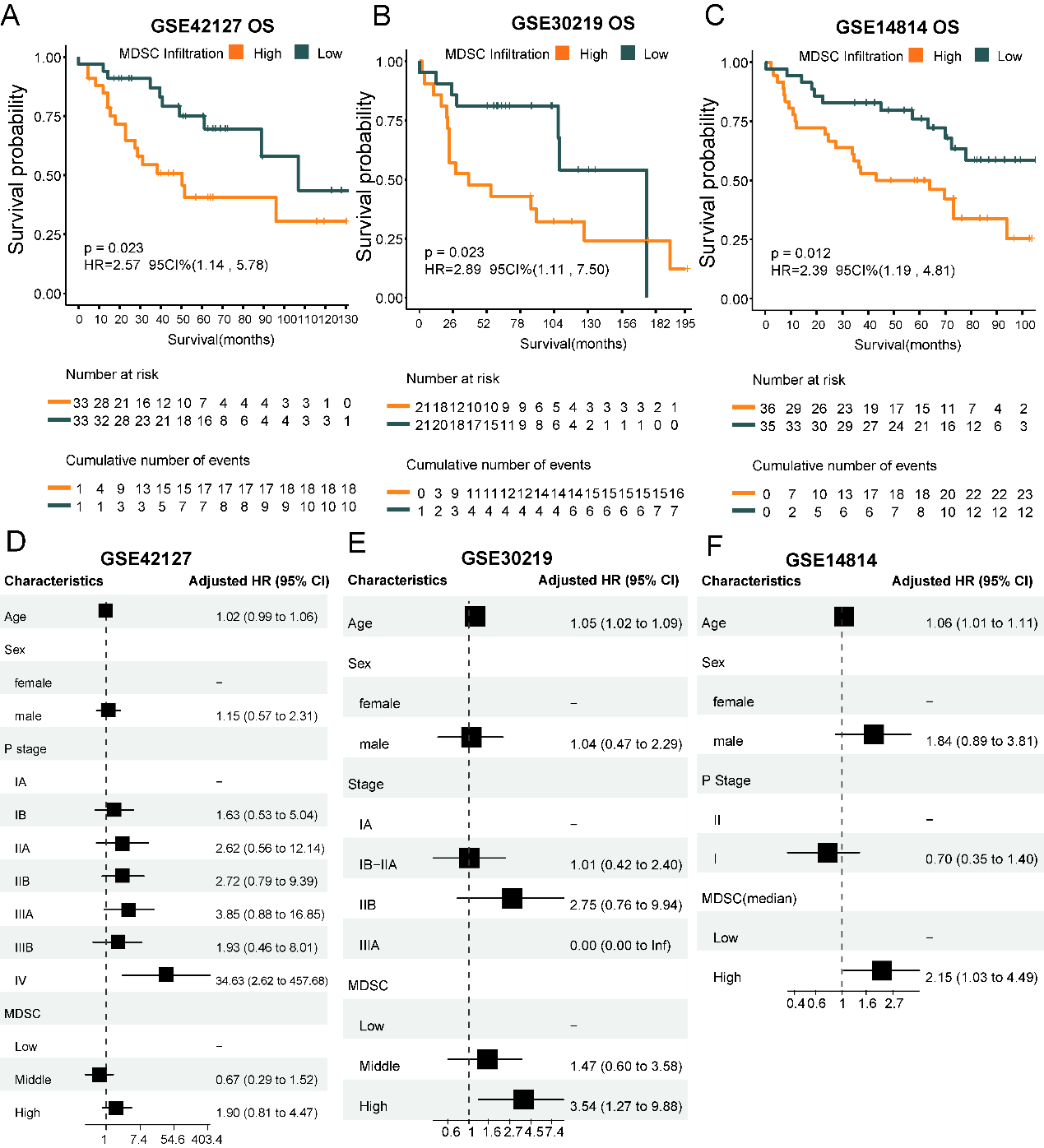
**

**Figure S2.** Survival analysis between groups with high and low MDSC infiltration in lung adenocarcinoma. **A-C:** Kaplan‒Meier survival curves between the high- and low-invasive groups in the GSE42127, GSE30219, and GSE14814 cohorts; the log-rank test was used to test the significance of survival rates between groups. **D-F:** Multivariate Cox regression analysis of the GSE42127, GSE30219, and GSE14814 lung adenocarcinoma cohorts. Mu-Cox multivariate Cox regression analysis; HR: hazard ratio; 95% CI: 95% confidence interval.

**Figure S3**

**
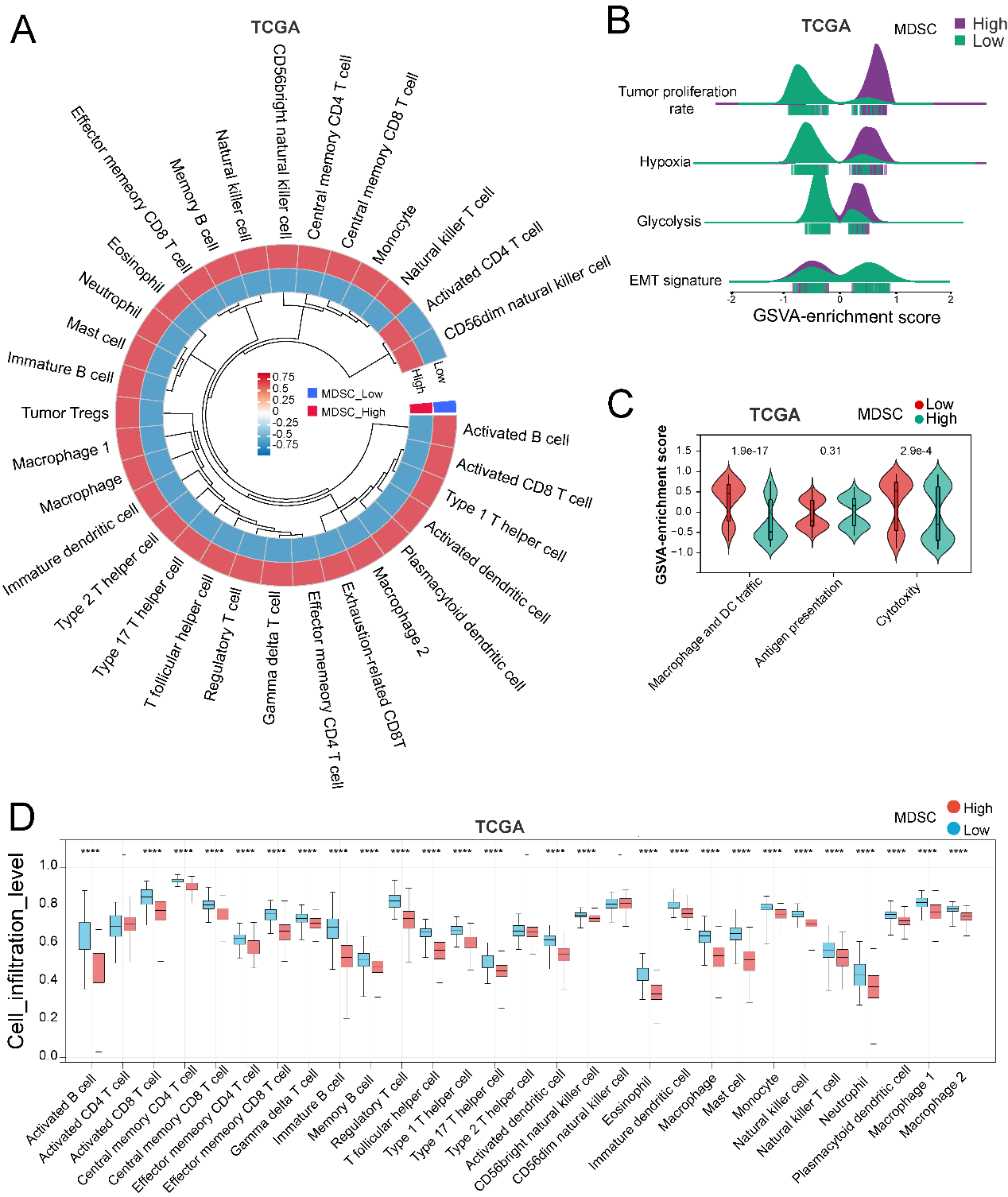
**

**Figure S3.** Assessment of antitumour immune responses between MDSCs with high and low infiltration in the TCGA cohort. **A:** Correlation heatmap of circular lines between MDSCs and other immune cell infiltration levels in lung adenocarcinoma. **B:** Ridge plot of differences in the biological behavior of tumor cells between MDSCs with high and low infiltration. **C:** Violin plot of the differences in antitumour immune responses between MDSCs with high and low infiltration. D: Box plot of the differences in the infiltration levels of immune cells between MDSCs with high and low infiltration. -, no significant difference; *, P < 0.05; **, P < 0.01; ***, P < 0.001; ****, P < 0.0001.

**Figure S4**

**
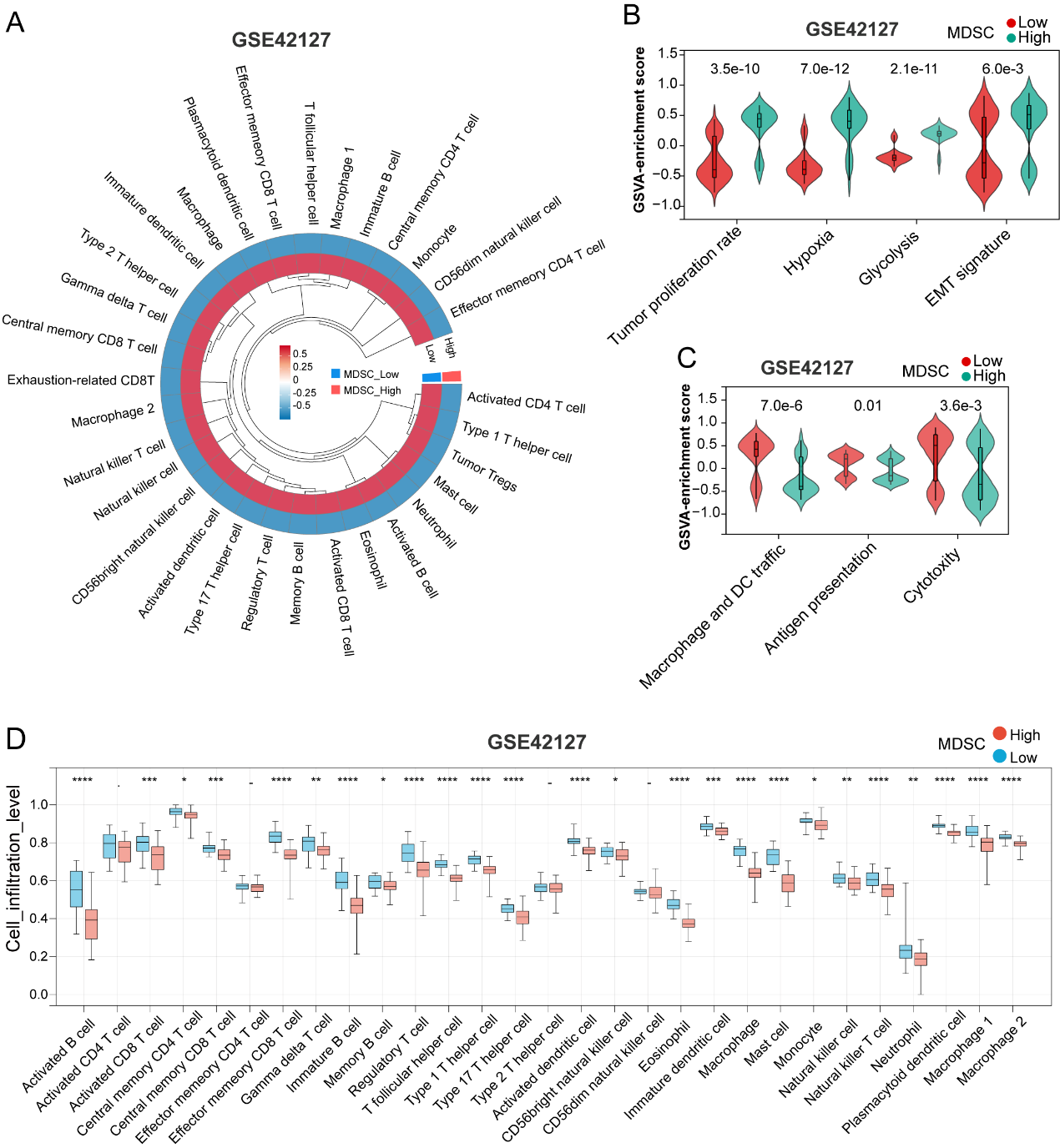
**

**Figure S4.** Assessment of antitumour immune responses between MDSCs with high and low infiltration in the GSE42127 cohort; **A:** Correlation heatmap of circular lines between MDSCs and other immune cell infiltration levels in lung adenocarcinoma. **B:** Violin plot of differences in the biological behavior of tumor cells between MDSCs with high and low infiltration. **C:** Violin plot of the differences in antitumour immune responses between MDSCs with high and low infiltration. D: Box plot of the differences in the infiltration levels of immune cells between MDSCs with high and low infiltration. -, no significant difference; *, P < 0.05; **, P < 0.01; ***, P < 0.001; ****, P < 0.0001.

**Figure S5**

**
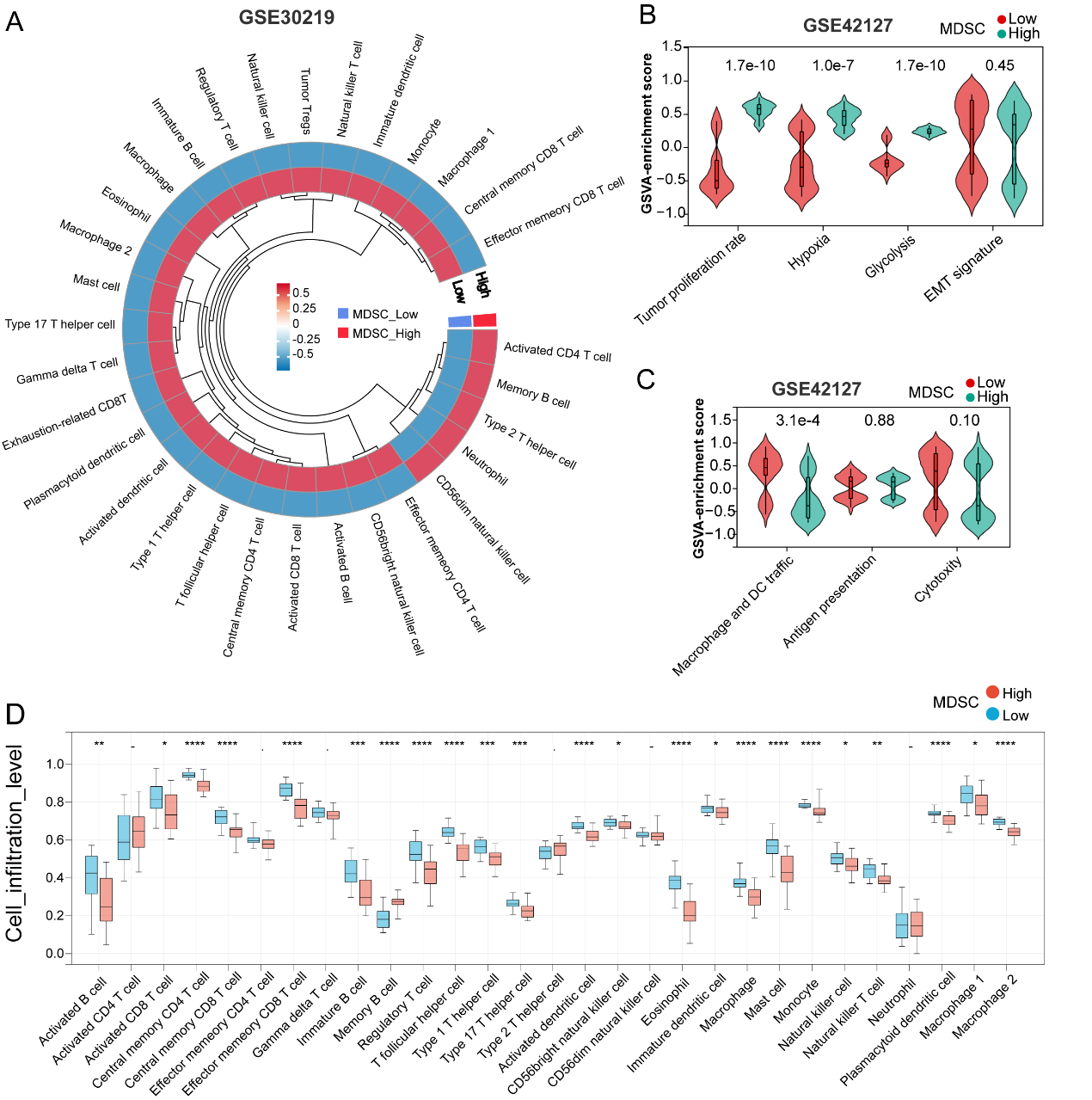
**

**Figure S5.** Assessment of antitumour immune responses between MDSCs with high and low infiltration in the GSE30219 cohort; **A:** Correlation heatmap of circular lines between MDSCs and other immune cell infiltration levels in lung adenocarcinoma. **B:** Violin plot of differences in the biological behavior of tumor cells between MDSCs with high and low infiltration. **C:** Violin plot of the differences in antitumour immune responses between MDSCs with high and low infiltration. D: Box plot of the differences in the infiltration levels of immune cells between MDSCs with high and low infiltration. -, no significant difference; *, P < 0.05; **, P < 0.01; ***, P < 0.001; ****, P < 0.0001.

**Figure S6**

**
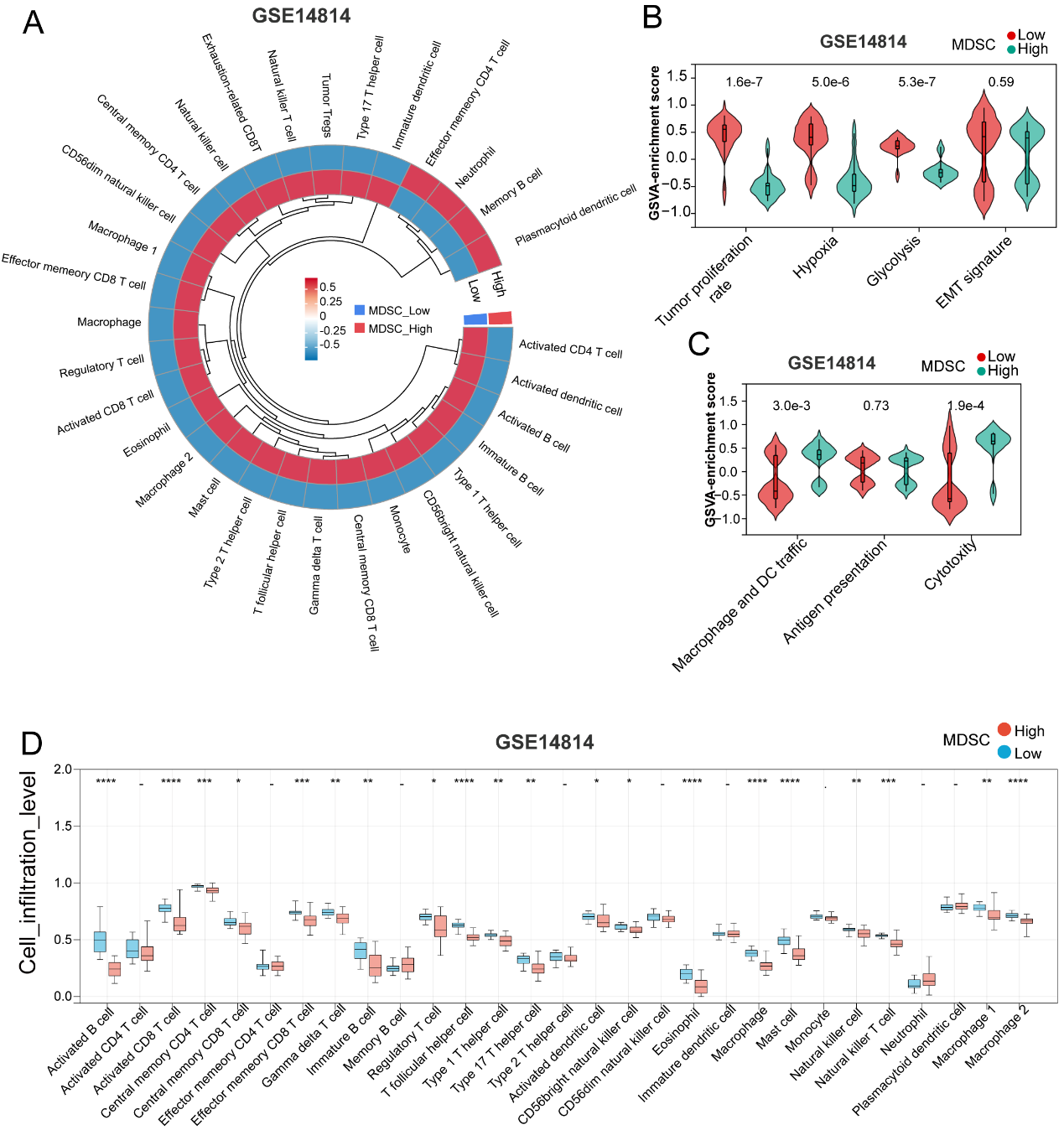
**

**Figure S6.** Assessment of antitumour immune responses between MDSCs with high and low infiltration in the GSE14814 cohort; **A:** Correlation heatmap of circular lines between MDSCs and other immune cell infiltration levels in lung adenocarcinoma. **B:** Violin plot of differences in the biological behavior of tumor cells between MDSCs with high and low infiltration. **C:** Violin plot of the differences in antitumour immune responses between MDSCs with high and low infiltration. D: Box plot of the differences in the infiltration levels of immune cells between MDSCs with high and low infiltration. -, no significant difference; *, P < 0.05; **, P < 0.01; ***, P < 0.001; ****, P < 0.0001.

**Figure S7**

**
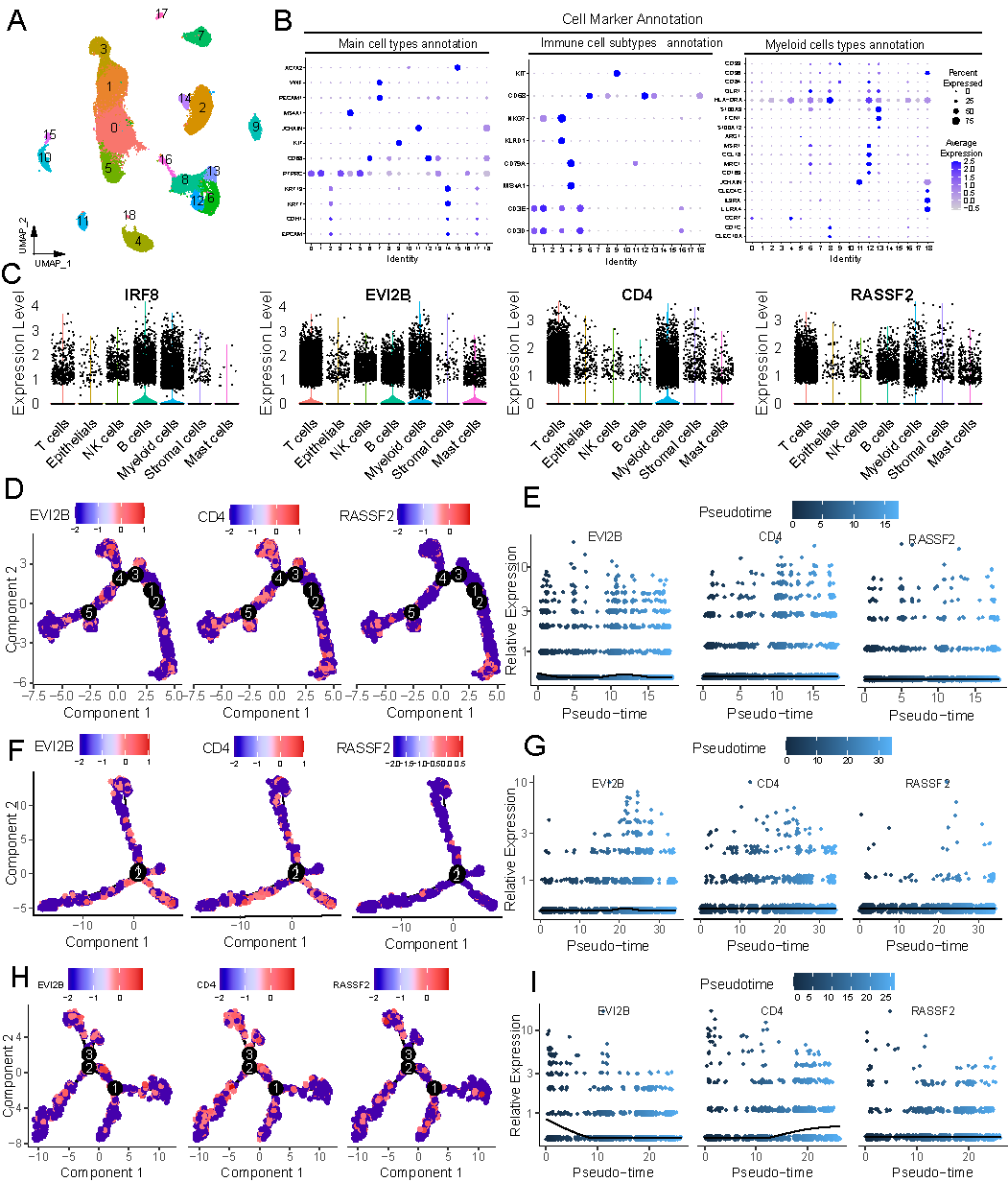
**

**Figure S7.** Single-cell RNA-Seq analysis of lung adenocarcinoma; **A:** UMAP after data filtering and quality control. **B:** Mean expression and percentage expression of selected marker genes in cell types. **C:** Violin plot of IRF8, EVI2B, RASSF2, and CD4 expression in the main cell subsets. **D:** Trajectories of EVI2B, RASSF2, and CD4 distribution in myeloid cell subsets. **E:** Map of EVI2B, RASSF2, and CD4 distribution in myeloid cell subsets. **F:** Trajectories of EVI2B, RASSF2, and CD4 distribution in MDSC populations. **G:** Map of EVI2B, RASSF2, and CD4 distribution in MDSC populations. **H:** Distribution of EVI2B, RASSF2, and CD4 trajectories in myeloid dendritic cell populations. **I:** Map of EVI2B, RASSF2, and CD4 distribution in myeloid dendritic cell populations.

**Figure S8**

**
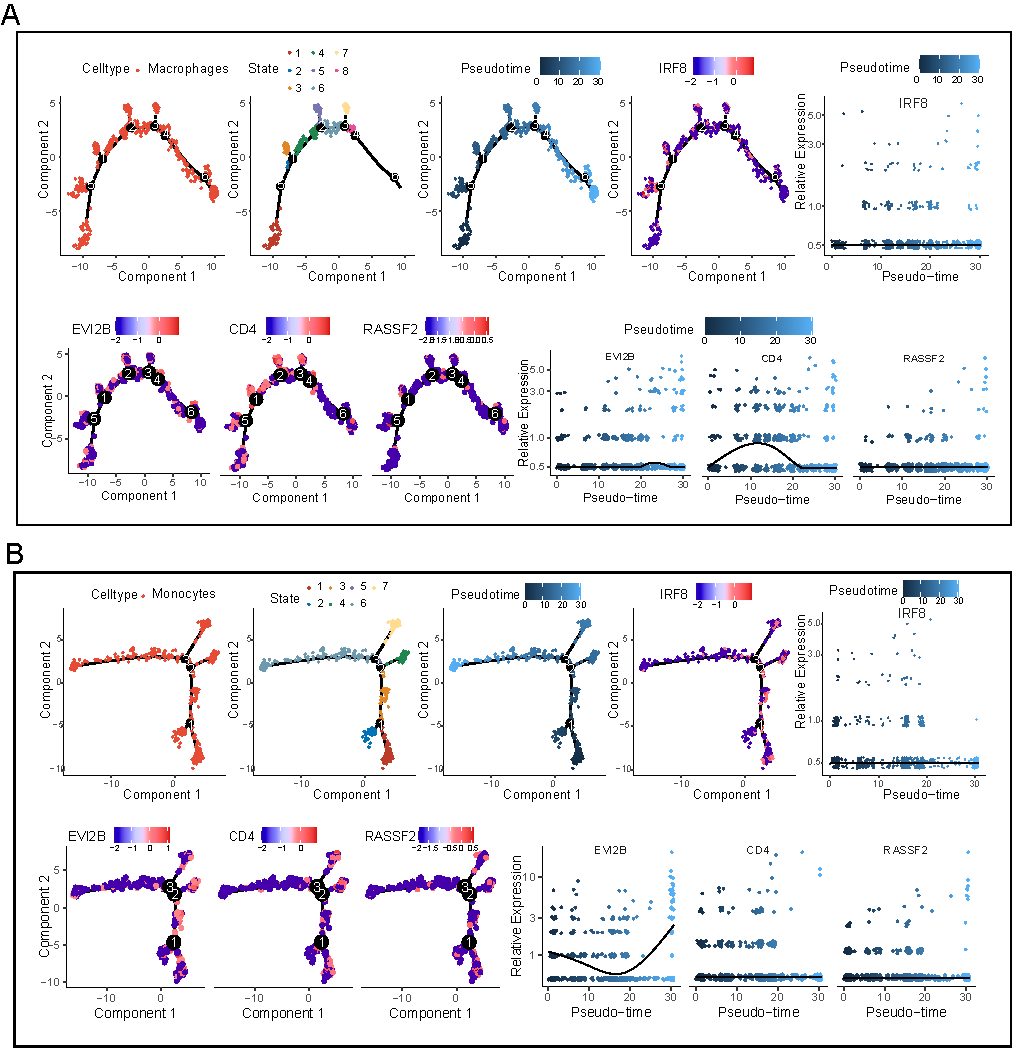
**

**Figure S8.** Single-cell RNA-Seq Analysis of Lung Adenocarcinoma; **A:** Temporal trajectories of macrophages and distribution of IRF8, EVI2B, RASSF2, and CD4. **B:** Temporal trajectories of monocytes and distribution of IRF8, EVI2B, RASSF2, and CD4.

**Figure S9**

**
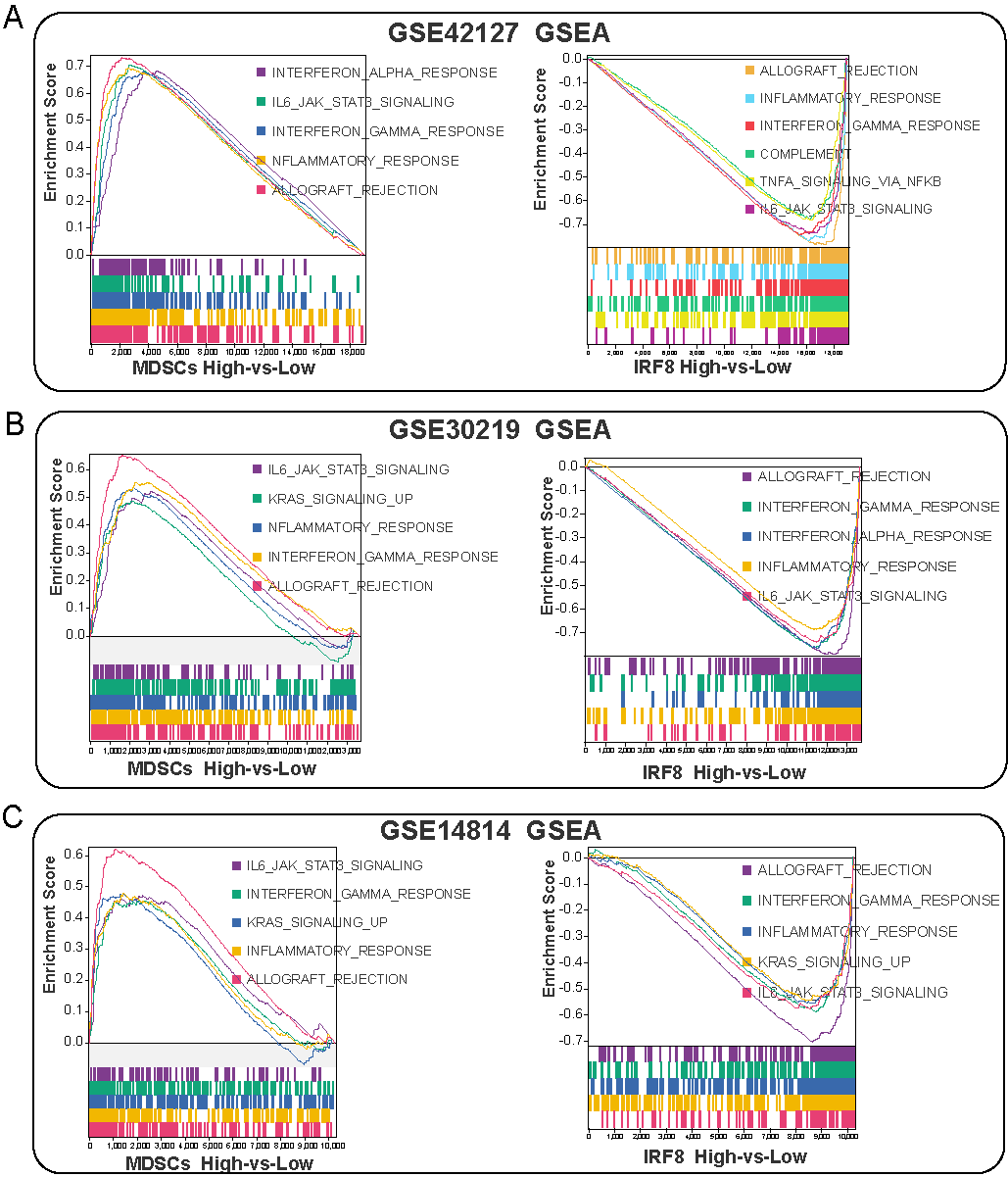
**

**Figure S9.** Identification of signalling pathways regulating the differentiation of MDSCs **A:** GSEA between groups with high and low MDSC infiltration and groups with high and low IRF8 expression in the GSE42127 cohort; **B:** GSEA between groups with high and low MDSC infiltration and groups with high and low IRF8 expression in the GSE30219 cohort; **C:** GSEA between groups with high and low MDSC infiltration and groups with high and low IRF8 expression in the GSE14814 cohort.
